# Disparities in stillbirth rates according to municipal deprivation levels: a nationwide study in Brazil

**DOI:** 10.1186/s12884-025-08258-9

**Published:** 2025-10-27

**Authors:** Ismina Papadhima, Lays Janaina Prazeres Marques, Elzo Pereira Pinto, Hannah Blencowe, Marcia Furquim de Almeida, Maria Yury Ichihara, Mauricio L. Barreto, Enny S. Paixao

**Affiliations:** 1 Schulich School of Medicine & Dentistry, Western University, London, ON Canada; 2 Department of Epidemiology, School of Public Health, University of São Paulo, São Paulo, Brazil; 3 Centre for Data and Knowledge Integration for Health (CIDACS), Fundação Oswaldo Cruz, Salvador, Brazil; 4 Faculty of Epidemiology and Population Health, London School of Hygiene and Tropical Medicine, London, UK

**Keywords:** Stillbirths, Fetal mortality, Social deprivation, Poverty, Time series studies

## Abstract

**Background:**

Investigating the relationship between stillbirth and deprivation is essential to guide healthcare improvements, as evidence is scarce in LMIC contexts. This study estimated the stillbirth rate (SBR) and the odds ratios (OR) of stillbirth in Brazil’s municipal deprivation context.

**Methods:**

This observational study included births in Brazil registered in the SINASC and SIM databases, employing two epidemiological designs. First, a cross-sectional analysis assessed the association between stillbirths and municipal deprivation, using data from January 1 to December 31, 2018. Logistic regression was used to estimate OR for stillbirths across deprivation levels, adjusting for sociodemographic, gestational, and fetal variables, with 95% confidence intervals. Deprivation was classified into quintiles based on Brazilian deprivation index (IBP) levels 1 to 5. Second, an ecological analysis examined time trends in SBR by deprivation level from January 1, 2000, to December 31, 2018. Time trends in SBR from 2000 to 2018 were analyzed using Prais-Winsten regression, both overall and stratified by IBP level.

**Results:**

In 2018, the OR of stillbirth, including both antepartum and intrapartum cases, increased with higher levels of deprivation. Compared to the least deprived areas, level 2 had a 9% greater OR of stillbirth (95%CI: 1.03–1.15), level 3 had a 30% higher OR (95% CI: 1.23–1.27), level 4 showed a 34% increase (95%CI: 1.27–1.41), and level 5 had the highest OR, with a 68% increase (95%CI: 1.60–1.77). From 2000 to 2018, SBR in Brazil declined by 1.1% per year (*p* < 0.001). Significant declines were observed across deprivation levels 1 (-1.6% per year; *p* < 0.001) to 4 (-1.5% per year; *p* < 0.001), while level 5 showed persistently high stillbirth rates with no significant improvement (*p* ≥ 0.05).

**Conclusion:**

These results highlight the stark inequalities in stillbirth chances across Brazil. Targeted action is needed to close the gap in the most deprived municipalities and reduce stillbirth rates and perinatal health disparities.

## Introduction

Stillbirth is a public health issue that is often neglected on the global and national scale [1]. Approximately 1.9 million stillbirths occurred worldwide in 2021, with 98% of these cases concentrated in low-income countries (LIC) and middle-income countries (MIC) [2, 3]. Low socioeconomic status, inadequate maternal healthcare access, and poor quality of care received during pregnancy and birth are important contributors to high stillbirth rates [4]. Addressing these disparities is essential to reducing preventable stillbirths and closing equity gaps [5].

The global stillbirth rate has decreased by 35%, from 21.3 to 13.9 per 1,000 births between 2000 and 2021[2]. Between 2000 and 2019, stillbirth rates decreased across all income groups: from 29.5 to 22.7 per 1,000 births in LIC, from 28.0 to 17.1 in lower-MIC, from 13.4 to 7.0 in upper-MIC, and from 3.9 to 3.0 in high-income countries [1]. In Brazil, the stillbirth rate declined from 10.1 to 7.0 per 1,000 births during the same period, reflecting a 30.7% reduction[2]. This decrease has been markedly slower than maternal and neonatal mortality [6, 7]. Furthermore, the national stillbirth rate masks the disparities that exist at the regional and municipality levels in Brazil. The country’s poorest region, the Northeast, had the highest rate at 12.1, while 7.7 was found in 2012 in the richest region, the South [8].

Although regional rates may provide a more accurate representation of stillbirth trends in Brazil, the significant socioeconomic differences at more localized levels, such as municipalities and neighborhoods, call for a more detailed analysis. However, stillbirth remains underexplored at this level, despite the need to investigate these variations to better understand its determinants and inequalities [8]. There are previous studies that analyse social deprivation and stillbirths in Brazil [9] and some cities in the Southeast, Northeast, and South regions [10–12]. However, each study adopted a specific local index of social deprivation, which implies limitations in comparing results. Therefore, investigating stillbirths at a disaggregated level is important to consider differences in small-area socioeconomic conditions and highlight areas for improvement in perinatal healthcare access and quality. Furthermore, no studies have quantified changes in the stillbirth risk over time by level of material deprivation in Brazil. Currently, it is unclear whether the national decline in the stillbirth risk was similar among municipalities with different deprivation levels. Bridging this knowledge gap may be instrumental in identifying areas that require more support and creating tailored approaches to reducing stillbirth in these highly affected areas.

This study investigates the association between stillbirth risk and municipal deprivation in Brazil. We will also examine temporal trends in the overall stillbirth rates by deprivation level from 2000 to 2018.

## Methods

### Study design and data sources

In this observational study, all births in Brazil registered in the SINASC and SIM databases were eligible, employing two epidemiological designs. First, a cross-sectional analysis assessed the risk of stillbirth by IBP scores, using individual-level data from January 1 to December 31, 2018. Second, an ecological analysis examined time trends in stillbirth rates (SBR) by deprivation levels from January 1, 2000, to December 31, 2018. Both designs relied on routinely collected health data from Brazil’s Mortality Information System (SIM) and Live Birth Information System (SINASC), maintained by the Ministry of Health. These systems compile nationwide data from standardized legal forms completed by physicians for deaths and by healthcare providers for live births. SIM and SINASC capture over 97% of deaths and births across Brazil [13].

Data on stillbirths were extracted from SIM, while live birth information was obtained from SINASC. For the cross-sectional analysis, de-identified individual-level data were publicly available on the Brazilian Ministry of Health’s website (https://datasus.saude.gov.br/). For the ecological analysis, data were obtained at the municipal level. Since all births in Brazil during the study period were elegible, a sample size calculation was not required.

### Study population and definitions

In accordance with the 11th revision of the International Classification of Diseases (ICD-11), stillbirth was defined as the delivery of a fetus with no signs of life at 22 weeks of gestation or more [14]. Fetal deaths were excluded from the study if they occurred before 22 weeks of gestation. The outcome was further categorized into antepartum and intrapartum stillbirths. Antepartum stillbirths were defined as fetal deaths occurring before the onset of labor. If the vital status of the fetus at the onset of labor is unknown, it is considered an antepartum fetus if signs of maceration are present at the time of delivery. Intrapartum stillbirths refer to fetuses that die during labor. If the vital status of the fetus at the onset of labor is unknown, it is considered to be intrapartum if there is a fresh skin appearance or no signs of maceration at the time of delivery [14].

Municipality deprivation of the maternal place of residence was assessed for each studied birth. Deprivation scores were obtained from the Brazilian Deprivation Index (IBP), a measure of material deprivation for each municipality [15]. The IBP was calculated by combinig z-score of variables selected to reflect the different domains of wealth, education and housing and include: (1) percent of households with per capita income below ½ minimum Brazilian wage; (2) percent of people not literate, aged 7+; and (3) average of percent of people with inadequate access to sewage, water, garbage collection and no toilet, using data from the 2010 Brazilian Population Census.

The z-score *z* for a variable *x* is calculated using the formula z = (x − µ)/sd, where the mean µ and standard deviation sd for the individual indicators are population weighted. The calculations were done using the zscore function in the R package SocEpi. The z-scores were weighted equally by simply summing them into a single deprivation measure.

### Study variables and data processing

Covariates were described, including maternal age (< 20, 20–24, 25–29, 30–34, 35–39, ≥ 40), maternal education (none, 1–3 years, 4–7 years, 8–11 years, ≥ 12 years), sex of the newborn (male, female), number of babies in the pregnancy (1, ≥ 2), gestational age at delivery (22–27 weeks, 28–31 weeks, 32–36 weeks, 37–41 weeks, ≥ 42 weeks), birthweight (< 1000 g, 1000–1499 g, 1500–2499 g, 2500–3999 g, ≥ 4000 g), history of previous fetal deaths (no, yes), and history of previous live births (no, yes). The percentage of missing data was reported for each variable, and complete case analyses were performed. The significance of differences in the frequencies of distribution between stillbirths and live births was verified using the Pearson Chi-square (χ^2^) test (*p* < 0.05). The municipality deprivation was grouped into quintiles, whereby a score of one corresponded to the lowest levels of deprivation, and a score of five represented the highest levels of deprivation.

### Statistical analyses

Using data from 2018, descriptive analyses by covariates were performed for stillbirths and live births. Additionally, the stillbirth rate stratified by stillbirth status (antepartum and intrapartum) was reported per thousand births (/1000 births) for the IBP score quintiles and the total. The stillbirth rate was calculated as the number of stillbirths divided by the total number of births, obtained by summing live births and stillbirths, per thousand births.

To estimate the odds ratio of stillbirth by IBP scores, logistic regression analyses were adopted with 95% confidence intervals (95% CI). The outcome variable was stillbirth (yes/no). We performed a complete case analysis adjusted for maternal age, maternal education, sex of the baby, number of babies in the pregnancy, gestational age at delivery, birth weight, history of previous fetal deaths, and history of previous live births.

Prais-Winsten generalized linear regression was used to estimate the time trends in the stillbirth rate by deprivation levels quintile between 2000 and 2018. The dependent variable was the logarithm of the stillbirth rate in Brazil and IBP scores, and the independent variable was the calendar years. The Durbin-Watson statistic was calculated to test for autocorrelation by comparing it with the two sets of critical values: the lower bound (dL) and upper bound (dU), both determined at a 5% significance level (dL = 1.180, dU = 1.401).

The Annual Percent Change (APC) was obtained by: APC = [−1 + 10^β [1]^]0.100 and the respective 95% confidence intervals: 95%CI = [−1 + 10^β1min^]0.100; [−1 + 10^β1max^]0.100. It was verified whether the trend of the risk was stationary (*p* ≥ 0.05), decreasing (*p* < 0.05 and β1 negative), or increasing (*p* < 0.05 and β1 positive) [16]. P-values were obtained with the Wald test. The significance level adopted in the study was 5%. Data were tabulated and analyzed on an Excel spreadsheet, STATA MP 14.0 (StataCorp), and the R software (The R Project for Statistical Computing).

### Patient and public involvement

The patients and the public were not involved in the design, conduct, or reporting of our research.

## Results

In 2018, 28,606 stillbirths and 2,944,932 live births were registered in Brazil. The stillbirth rate (per 1000 births) was highest among IBP level 5 (11.8/1000 births), women aged 40 or older (16.8/1000 births), pregnancies with two or more fetuses (23.8/1000 births), history of previous fetal death (20.0/1000 births), and among male fetus (9.8/1000 births). The stillbirth rate was the highest for the lowest category level of maternal education, gestational length, and birthweight, decreasing markedly as category-level values increased (Table 1).

**Table 1:** Characteristics of live births and stillbirths, stillbirth rate in Brazil in 2018 (*N* = 2,973,538)

Characteristics	Live births *N* (%)	Stillbirths *N* (%)	Stillbirth rate^b^	*p*-value^c^
IBP Score				
1	487,258 (16.6)	3,660 (12.9)	7.5	< 0.001
2	526,660 (17.9)	4,451 (15.6)	8.4	
3	591,145 (20.1)	5,668 (19.9)	9.5	
4	621,952 (21.1)	6,127 (21.5)	9.8	
5	717,157 (24.4)	8,546 (30.0)	11.8	
Missing^a^	760 (0.0)	154 (0.5)		
Maternal age (years)				
< 20	456,128 (15.5)	4,271 (16.1)	9.3	< 0.001
20–24	723,352 (24.6)	6,126 (23.2)	8.4	
25–29	696,559 (23.7)	5,554 (21.0)	7.9	
30–34	611,715 (20.8)	5,092 (19.3)	8.3	
35–39	365,814 (12.4)	3,845 (14.5)	10.4	
≥ 40	91,295 (3.1)	1,563 (5.9)	16.8	
Missing^a^	69 (0.0)	2,155 (7.5)		
Maternal education level (years)				
0	12,226 (0.4)	856 (3.6)	65.4	< 0.001
1–3	53,925 (1.9)	1,505 (6.4)	27.2	
4–7	439,971 (15.2)	5,641 (24.0)	12.7	
8–11	1,782,145 (61.4)	12,377 (52.6)	6.9	
≥ 12	613,793 (21.2)	3,148 (13.4)	5.1	
Missing^a^	42,872 (1.6)	5,079 (17.8)		
Number of babies in pregnancy				
1	2,876,110 (97.8)	26,052 (94.4)	9.0	< 0.001
≥ 2	63,417 (2.2)	1,545 (5.6)	23.8	
Missing^a^	5,405 (0.2)	1,009 (3.5)	9.0	
Sex of baby				
Male	1,508,595 (51.2)	14,854 (53.1)	9.8	< 0.001
Female	1,435,840 (48.8)	13,125 (46.9)	9.1	
Missing^a^	497 (0.0)	627 (2.2)		
Gestational age at delivery (weeks)				
22–27	16,035 (0.6)	6,841 (26.0)	299.0	< 0.001
28–31	29,741 (1.0)	4,932 (18.7)	142.2	
32–36	277,900 (9.6)	7,492 (28.6)	26.3	
37–41	2,502,360 (86.2)	6,938 (26.4)	2.8	
≥ 42	75,929 (2.6)	131 (0.1)	1.7	
Missing^a^	42,967 (1.5)	2,272 (7.9)		
Birth weight (g)				
< 1,000	16,993 (0.6)	7,838 (30.0)	315.7	< 0.001
1,000–1,499	21,871 (0.7)	4,103 (15.7)	158.0	
1,500-2,499	209,913 (7.1)	6,947 (26.6)	32.0	
2,500-3,999	2,538,932 (86.3)	6,448 (24.7)	2.5	
≥ 4,000	154,637 (5.3)	819 (3.1)	5.3	
Missing^a^	2,586 (0.1)	2,451 (8.6)		
History of previous fetal deaths				
No	2,265,691 (80.9)	14,578 (57.2)	6.4	< 0.001
Yes	534,877 (19.1)	10,905 (42.8)	20.0	
Missing^a^	144,364 (4.9)	3,123 (10.9)		
History of previous live births				
No	1,196,101 (41.9)	10,997 (41.9)	9.1	0.17
Yes	1,657,604 (58.1)	15,225 (58.1)	9.1	
Missing^a^	91,227 (3.1)	2,384 (8.3)		

^a^Percentages for missing data are calculated as a proportion of total live births and stillbirths. ^b^per 1000 births. ^c^Pearson’s chi-square test

**Fig. 1:**
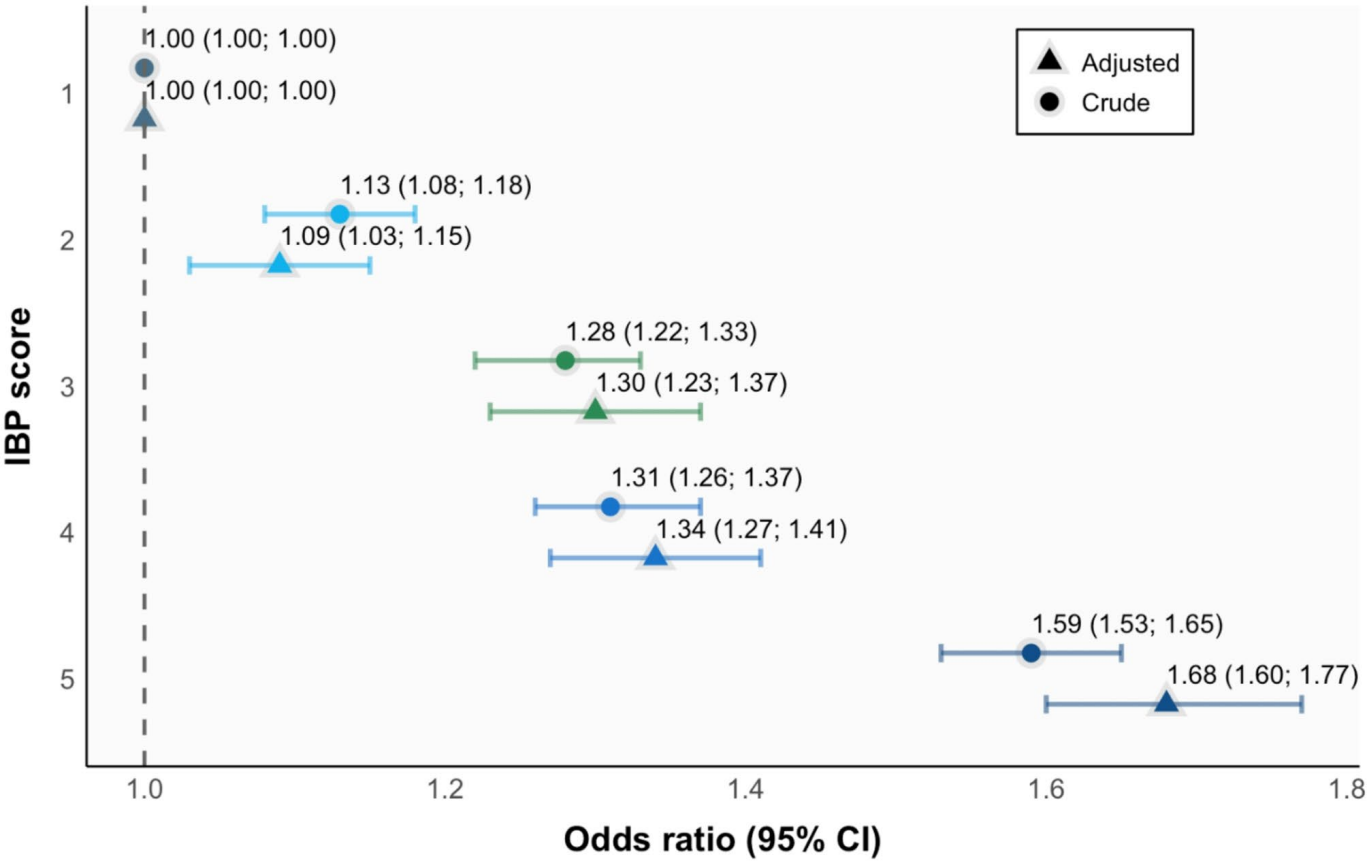
Odds ratio adjusted and crude for stillbirths according to IBP score 1 (less deprived) to 5 (most deprived) in Brazil, 2018. Note: 95%CI − 95% confidence interval. IBP – Brazilian deprivation index. OR adjusted for maternal age, maternal education, sex of the baby, number of babies in the pregnancy, gestational age, birth weight, history of previous fetal deaths, and history of previous live births

In 2018, stillbirth rates increased progressively with higher levels of deprivation, being lowest at level 1 and highest at level 5, ranging from 7.5 to 11.8 stillbirths/1000 births, respectively. The forest plot displays the adjusted odds ratios for stillbirth by IBP scores. Compared to the least deprived areas, level 2 had a 9% greater odds ratio of stillbirth (95% CI: 1.03–1.15), level 3 had a 30% higher odds ratio (95% CI: 1.23–1.27), level 4 showed a 34% increase (95% CI: 1.27–1.41), and level 5 had the highest odds ratio, with a 68% increase (95% CI: 1.60–1.77) (Fig. 1).

Most stillbirths were antepartum (*n* = 25,847; 90.4%), with a rate of 8.7 stillbirths/1000 births, contrasted with an intrapartum stillbirth rate of 0.4/1000 births (*n* = 1,229; 4.3%). The stillbirth timing information was absent for 1,530 (5.3%) stillbirths. Both antepartum and intrapartum stillbirth rates increased with higher levels of deprivation, with antepartum stillbirth rates ranging from 6.9 to 10.5 stillbirths/1000 births and intrapartum stillbirth rates from 0.3 to 0.6 stillbirths/1000 births between levels 1 and 5 (Table 2).

**Table 2:** Antepartum and intrapartum stillbirths and stillbirth rates on a national scale, and by deprivation level in Brazil in 2018 (*N* = 2,973,538)

Level	Antepartum stillbirths *N* (%)	Antepartum stillbirth rate^b^	Intrapartum stillbirths *N* (%)	Intrapartum stillbirth rate^b^
Total	25,847	8.7	1,229	0.4
IBP Score				
1	3,365 (13.1)	6.9	170 (13.9)	0.3
2	3,963 (15.4)	7.5	135 (11.0)	0.3
3	5,174 (20.1)	8.7	227 (18.5)	0.4
4	5,605 (21.8)	8.9	245 (19.9)	0.4
5	7,651 (29.7)	10.5	450 (36.7)	0.6
Missing^a^	89 (0.3)		2 (0.2)	

*p* < 0.001 for all measures of stillbirth rate

^a^Percentages for missing data for IBP Scores are calculated as a proportion of antepartum stillbirths, and intrapartum stillbirths. ^b^Per 1000 births

**Fig. 2:**
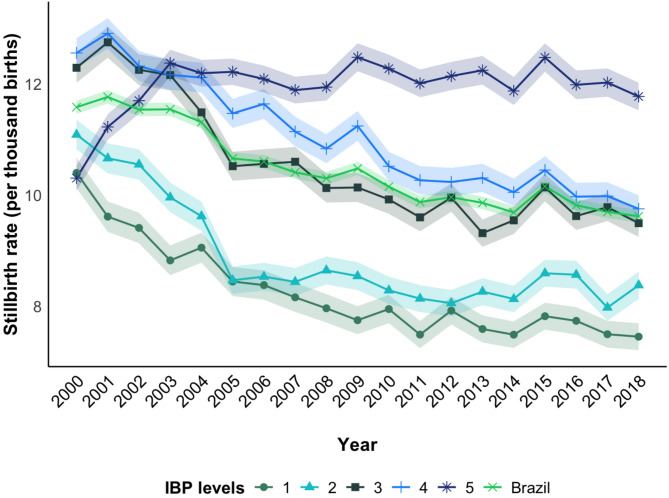
Time trends of total stillbirth rate and by level of deprivation in Brazil from 2000 to 2018. Note: IBP – Brazilian deprivation index

From 2000 to 2018, 57,040,569 births occurred, of which 598,334 (1.0%) were stillbirths and 56,442,235 (99.0%) live births, recorded in the SIM and SINASC, respectively. The time series analysis revealed an annual overall reduction of 1.1% in the stillbirth rate and 1.6%, 1.5%, 1.5%, and 1.5% among deprivation levels 1, 2, 3, and 4, respectively. There is no evidence of a trend in the stillbirth rate in the period in the most deprived group (IBP level 5) (APC = 0.4%; *p* = 0.095) (Table 3; Fig. 2).

**Table 3:** Prais-winsten regression estimates for the total stillbirth rate (per thousand births) and by IBP scores 1 (less deprived) to 5 (most deprived) in Brazil, 2000–2018

Level	Stillbirth rate																			APC (%)	95%CI	Trend	
	2000	2001	2002	2003	2004	2005	2006	2007	2008	2009	2010	2011	2012	2013	2014	2015	2016	2017	2018				
Total	11.5	11.7	11.5	11.6	11.3	10.7	10.6	10.4	10.3	10.5	10.2	9.9	10.0	9.9	9.7	10.2	9.8	9.7	9.6	−1.1	−1.41; −0.77		Decreasing^b^
IBP Scores																							
1	10.4	9.6	9.4	8.8	9.1	8.4	8.4	8.2	8.0	7.8	8.0	7.5	7.9	7.6	7.5	7.8	7.7	7.5	7.5	−1.6	−2.21; −1.03		Decreasing^b^
2	11.1	10.7	10.6	10.0	9.6	8.5	8.5	8.4	8.6	8.5	8.3	8.1	8.1	8.3	8.1	8.6	8.6	8.0	8.4	−1.5	−2.57; −0.45		Decreasing^c^
3	12.3	12.8	12.3	12.2	11.5	10.5	10.6	10.6	10.1	10.1	9.9	9.6	10.0	9.3	9.5	10.1	9.6	9.8	9.5	−1.5	−2.22; −0.84		Decreasing^b^
4	12.6	12.9	12.3	12.2	12.1	11.5	11.6	11.1	10.8	11.2	10.5	10.3	10.2	10.3	10.1	10.5	10.0	10.0	9.8	−1.5	−1.74; −1.26		Decreasing^b^
5	10.3	11.2	11.7	12.4	12.2	12.2	12.1	11.9	11.9	12.5	12.3	12.0	12.1	12.2	11.9	12.5	12.0	12.0	11.8	0.4	−0.08; 0.95		Stationary

^a^APC - annual percent change; ^b^*p*<0.001 and ^c^*p*<0.05 (Wald Test)

## Discussion

The risk of stillbirth increased progressively with higher levels of municipal deprivation. Even after adjusting for maternal, pregnancy, and birth outcome characteristics, the most deprived municipalities in 2018 had a 68% higher risk of stillbirth compared to the least deprived areas. Both antepartum and intrapartum stillbirth rates followed this pattern, with intrapartum stillbirth rates being particularly high in the most deprived areas. Time series analyses from 2000 to 2018 showed a decline in stillbirth rates across Brazil and deprivation levels 1 to 4. However, in areas with the highest level of deprivation, there was no evidence of any improvement in stillbirth rates during this period.

In 2018, Brazil’s national stillbirth rate was 9.6 per 1,000 births, below the target set by the Every Newborn Action Plan (ENAP) of 12 or fewer stillbirths per 1,000 births by 2030[17]. However, this rate varies substantially within the country. Although the most deprived region met the target with a stillbirth rate of 11.8, the least deprived region reported a markedly lower rate of 7.5, underscoring persistent inequities and substantial disparities in the quality of health care. The association between high deprivation and high stillbirth rates has been well-documented worldwide. On a global scale, 98% of all stillbirths in 2021 occurred among low- and middle-income countries (LMIC) [2]. Low socioeconomic status and inadequate perinatal care are often cited as two of the main risk factors for stillbirth in LMICs [18–21], including Brazil [22]. In high-income countries, stillbirth rates tend to be higher among marginalised and racialised groups, as well as highly deprived neighbourhoods [5, 23, 24].

Although many studies have identified socioeconomic status as a risk factor for stillbirth in Brazil [8–11], this is the first investigation to examine the association between municipality material deprivation and stillbirth rate on a national scale. The present study found a dose-response effect between IBP levels and stillbirth rates, showing that the higher the level of deprivation, the higher the stillbirth rate. This disparity in the stillbirth rate mirrors the inequitable stillbirth rates among Brazil’s five geopolitical regions, as described in the existing literature [8, 25]. In 2012, the poorest region, the Northeast, had the highest rate of 12.1 stillbirths/1000 births, while one of the richest regions, the South, had the lowest prevalence of 7.7/1000 births [8].

In agreement with the present findings, municipality deprivation is a well-established distal determinant of maternal-fetal health [26, 27]. Allik et al. validated the IBP, showing its correlation with health indicators and vulnerability at municipal levels. Disparities in IBP levels indicate structural inequities in maternal and child care [5, 23, 24]. Highly deprived areas often have less effective, safe, and timely healthcare, leading to higher stillbirth rates [5, 18, 24]. Lastly, municipality deprivation is associated with poor material living conditions, such as dangerous water sources, poor sanitation, poor hygiene practices, and unsafe neighbourhoods, which may lead to worse maternal health and, therefore, fetal compromise [5, 24].

Regarding stillbirth timing, this study found that antepartum and intrapartum stillbirths increased as deprivation levels increased. Previous Brazilian studies explored antepartum and intrapartum stillbirths in specific cities and states [12, 28–30]. However, this was the first known study to calculate national antepartum and intrapartum stillbirth rates. Our data showed that, although intrapartum stillbirth rates are low, the difference between municipalities with the highest and lowest deprivation index doubled, exceeding the disparity found in antepartum mortality, indicating clear differences in childbirth care based on deprivation levels. This pattern may be explained by the fact that the most deprived municipalities are predominantly located in rural areas of Brazil, as shown in previous studies [15], which can hinder timely access to childbirth care, as well as access to high-risk prenatal care. Higher levels of antepartum stillbirths suggest poor maternal health and inadequate care during pregnancy [18, 31]. In contrast, higher levels of intrapartum stillbirths suggest poor and delayed obstetric care, recognition of intrapartum complications, and management of compromised fetuses during labour [26, 31, 32].

From 2000 to 2018, the stillbirth rate among IBP levels 1 to 4 decreased, whereas IBP level 5 remained relatively steady. While the national and regional decline in stillbirth rates has been previously described [1, 8, 33], the present study explores yearly stillbirth rates by level of municipality deprivation in Brazil. The divergent stillbirth rate trend observed in IBP level 5 indicates that national socioeconomic development and maternal healthcare improvements over the past two decades may have been inequitable. Reductions in stillbirths at IBP levels 1 to 4 might be due to the implementation and expansion of various health policies and programs, which increased access to healthcare and improved women’s health, obstetric care quality, and access, and women’s sexual and reproductive health [33, 34]. In addition, there have been government interventions outside the health sector, including investments in education, improved water supply and sanitation, and decreased poverty and income inequality, which have contributed to reducing social disparities [34].

It is unclear why these interventions were comparatively less effective in reducing stillbirth rates in the most deprived municipality. It has been documented that municipalities in the highest quintiles of deprivation have a higher percentage of rural populations living in more remote areas[15],, far from large urban centers with high-complexity hospital care, which is especially crucial for high-risk pregnancies. In this context, the need to travel long distances, which represents an increased travel time, may explain why, despite the expansion of Primary Health Care services and social programs in Brazil, stillbirth rates have not decreased in the highest deprived municipalities [35, 36].

The current findings suggest that to reduce the stillbirth rate in Brazil, strategies tailored to specific municipality IBP levels must be implemented, including reducing national health and socioeconomic inequalities and improving access to high-complexity hospital care. Further investigation is required to explain this discrepant trend and evaluate the impact of government policies and interventions on the most deprived Brazilian municipalities to establish effective stillbirth reduction strategies.

This study has some limitations. The Brazilian Deprivation Index (IBP) used was developed based on data from the 2010 Brazilian Demographic Census, which may not fully capture socioeconomic changes throughout the entire study period (2000–2018). As more recent census data are not yet available, the use of the 2010 IBP could lead to misclassification of deprivation levels over time. Therefore, the findings should be interpreted with caution, and future research should consider updated deprivation measures as new data become available. We used the IBP score grouped by municipality level, which means we could not capture deprivation in smaller, more localized areas. However, it is worth noting that the municipality is a small administrative unit, including the health system. Additional study limitations may arise due to routine secondary data from SINASC and SIM. Although these information systems have significantly improved over time, the degree of incompleteness of some variables is still high, especially in SIM, mainly for sociodemographic characteristics, particularly maternal education in SIM, where missing data reached 18% We performed a complete cases analyses which may have influenced the estimates. Furthermore, the lack of SIM data on important risk factors, confounders, and mediating variables may have also affected study results and led to residual confounding bias.

This study analyzed Brazilian health registry data to explore national stillbirth rates and municipal deprivation. The results indicate that specific needs at each IBP level must be addressed to reduce stillbirth disparities effectively. Although stillbirth rates declined from 2000 to 2018 for IBP levels 1 to 4, they remained highest and stable for the most deprived municipalities. This suggests that while overall healthcare improvements and interventions may have contributed to reductions in stillbirth rates, these benefits were not uniformly experienced across all levels of deprivation. Based on our findings, policy actions should prioritize strengthening prenatal and childbirth care in socioeconomically deprived municipalities. This includes implementing tailored strategies to address their specific challenges, particularly by improving access to obstetric care to ensure safe childbirth. Although this study was conducted in Brazil, the results may be generalizable to other LMICs, where more deprived areas also experience higher stillbirth rates, reflecting global trends. Further research is needed to better understand the mechanisms driving these disparities to guide the development of more targeted interventions and health policies. Future studies should also explore the potential impact of the COVID-19 pandemic on the association between stillbirth and municipality-level inequalities.

## Data Availability

All data used in this study are publicly available.
